# Dr. Drake’s Letter

**Published:** 1844-08

**Authors:** Daniel Drake

**Affiliations:** New Orleans


### DR. DRAKE'S LETTER.

*To the Editors of the N. O. Medical Journal:*

Gentlemen:—You will greatly oblige me by inserting this note in the first number of your forthcoming Journal. I am in the 'lower country' as you know, collecting information on its topography, climate, and diseases; but of course shall not be able to visit every part of it, and must therefore make known my object to its physicians, through the press.

For many years I have had it in view to write a history of the prevalent maladies of the Mississippi Valley, but till within the last two, have been unable to make those personal observations which are indispensably necessary to the collection of the required facts and observations. In my visit to a part of the South-west, last spring, the courteous liberality of its medical gentlemen enabled me to amass much valuable *materiel;* but I need a great deal more, and as a visit to all parts is of course impracticable, I shall have to rely to some extent on this mode of soliciting additional contributions.

The remittent and intermittent fevers of the country, especially those of a malignant character, and the yellow fever of the cities and smaller towns, are objects of deepest interest. In reference to the whole, I ask for facts, rigidly observed and concisely recorded, without accompanying hypotheses or speculations of any kind, as the plan of my projected work excludes, I may say, almost every thing but facts. The circumstances under which autumnal fever assumes a malignant type, its treatment, and the pathological anatomy of its victims, are of the deepest interest. It is almost discreditable to us as a profession that, this fever, the great endemic of the United States, should be so little known by its anatomical characters. Essentially rural in its prevalence, it is to the physicians who practise in the country, that we must look for this, and indeed all portions of its history. Of yellow fever, the same remark might be made; but it is chiefly in New Orleans and Mobile, that its treatment and the lesions of structure which it produces, can be studied with success. When, however, it appears in villages the mode of its origin may be investigated, and facts collected that cannot fail to shed light on its cause. It frequently happens, that people go into the country with the *semina* of the fever in their systems, and are taken down. The degree in which the disease spreads from them, if it ever does, deserves the deepest attention. Thus village and country practitioners, not less than those of cities, may contribute to the settlement of the question which has so long divided the profession and agitated the community.

In the South, I find that *trismus nascentium* and *tetanus* both traumatic and idiopathic, are much more frequent than in the higher latitudes, and therefore deserve special attention.

There is reason to think, moreover, that uterine affections, both organic and functional, prevail more below, than above the latitude of 33°, and the study of them is of course a special duty of the physicians of the former.

On the other hand, the winter endemics, especially the phlegmasiæ of the lungs, with tubercular phthisis and scrofula, the natural group of spring and summer diseases, cholera morbus, cholera infantum, diarrhœa and dysentery; and the eruptive fevers, appear to be milder in these latitudes, than on the banks of the Ohio and the shores of the northern lakes, concerning the whole of which I am anxious to receive authentic information.

I have not, however, made these specifications with the design of limiting those who may feel inclined to afford assistance; for every new fact relative to the causes, symptoms, and treatment of all orders of the maladies of this region, will be gratefully received.

It is my intention to present, in separate chapters, an account of the physiology and diseases of the Negro and the Indian, compared with each other, and with the white man. For this purpose I have collected a considerable number of interesting observations, but wish for many more; and will be thankful to those who may have mingled much with the Indians, and those who practise on the cotton and sugar plantations, for all the facts they can supply, not only in relation to those races, but the families of mixed-blood Mulattoes, Quadroons, and Mestizoes. I am particularly anxious to acquire precise information concerning the results of the intermarriages of these races for several generations, as it has been lately affirmed, that after three or four generations they prove unprolific, and the races expire.

On my return to Louisville, at the opening of the next session of its Medical Institute, I hope to begin the arrangement of the facts I have collected, and will be pleased to receive contributions throughout the winter, and, indeed, to the end of the following summer. If gentlemen should at any time prefer to publish their observations, and for that purpose should send them to you, or to the editors of the Western Journal of Medicine and Surgery, in Louisville, they will be as available to my object as if sent to me in manuscript.

In conclusion, gentlemen, suffer me to express the hope that your laudable enterprize may be extensively patronized. Notwithstanding so many Journals are published in the United States, I have had opportunities of knowing that there are physicians in every part of the West and South, who do not receive one, although all who practise medicine should take at least two, if they would keep up with the improvements in the profession.

With respect,

I am your very ob't serv't,

DANIEL DRAKE, M.D.

New Orleans, May 9th, 1844.

